# Insights into the host-pathogen interaction: *C. albicans* manipulation of macrophage pyroptosis

**DOI:** 10.15698/mic2018.12.662

**Published:** 2018-11-12

**Authors:** Teresa R. O’Meara, Leah E. Cowen

**Affiliations:** 1 Department of Molecular Genetics, University of Toronto, Toronto, Ontario, Canada

**Keywords:** fungi, C. albicans, pyroptosis, innate immune responses, macrophage, inflammasome, cell wall.

## Abstract

The innate immune system is the first defense against invasive fungal infections, including those caused by *Candida albicans*. Although *C. albicans* can exist as a commensal, it can also cause systemic or mucosal infections, especially when the innate immune system is impaired. A key aspect of the interaction between *C. albicans* and innate immune cells is the ability of *C. albicans* to induce macrophage pyroptosis, an inflammatory cell death program. The induction of pyroptosis is temporally coupled to a morphological transition between yeast and filamentous growth. However, the relationship between fungal morphogenesis and activation of macrophage pyroptosis is complex. Although most *C. albicans* mutants with defects in filamentation are also unable to induce macrophage pyroptosis, filamentation is neither necessary nor sufficient for activation of pyroptosis. In our study [O’Meara et al., 2018 mBio], we set out to map the genetic circuitry in both the fungus and the host macrophage that leads to pyroptosis, and determine the impact of altered pyroptosis on infection. We identified 98 *C. albicans* genes that were dispensable for filamentation in the macrophage but important for enabling the fungus to activate macrophage pyroptosis. Using these mutants, we demonstrated that pyroptosis is required for robust neutrophil accumulation at the site of *C. albicans* infection. We also showed that, in contrast to previous work, inflammasome priming and activation can be decoupled in the response to *C. albicans* infection, and that phagolysosomal rupture is not the inflammasome activating signal. Our work provides the most comprehensive analysis of *C. albicans* interactions with host cells to date, and reveals new factors governing the outcomes of this interaction.

*C. albicans* is a human fungal pathogen that can also exist as a normal member of the healthy human mucosal microbiota. The ‘pathogenic potential’ of this microbe to cause disease depends on both the fungal virulence factors and the host immune responses. Unlike many other fungi that can cause disease in patients with defects in adaptive immune responses, *C. albicans* dissemination to the bloodstream and systemic infection is primarily observed in patients with defects in innate immune responses. As part of the interaction with innate immune cells, *C. albicans* can induce pyroptosis, an inflammatory programmed cell death that depends on the NLRP3, ASC, and caspase-1 proteins. These proteins form an inflammasome protein complex that activates caspase-1, allowing it to cleave gasdermin D, resulting in a gasdermin fragment that can form pores in the host cell membrane. The activation of the NLRP3 inflammasome can occur from many signals, but relatively little is known about how fungi can activate this process.

Functional genomics is a powerful approach to examine gene function and identify the genes governing this hostpathogen interface. We developed an imaging-based screen to examine the interaction between *C. albicans* and macrophages, with a focus on genes that are required for the induction of pyroptosis. When infected with wild-type *C. albicans*, macrophages will take up propidium iodide as a marker of cell death. By using high-throughput microscopy, we could simultaneously measure host cell death and the levels of filamentation for all of the 2356 GRACE tetracycline-repressible *C. albicans* mutants. It is important to assay filamentation during infection, as filament-inducing conditions *in vitro* may not accurately predict phenotypes during co-culture with host cells. We then used a more specific marker of inflammasome activation—the formation of an ASC speck—to identify *C. albicans* mutants with a defect in inducing pyroptosis. The 98 *C. albicans* genes that our screen identified as important for activation of pyroptosis gave us unprecedented power to examine the host-pathogen interface.

There are multiple stages of the macrophage-*Candida* interaction that need to occur for pyroptosis to be activated (Figure 1). The host cell must recognize and phagocytize the fungal cell, while the fungal cell must sense and respond to entry into the host cell and expose the trigger for pyroptosis. Additionally, the host cell must prime and activate the inflammasome. We identified mutants defective at each of these stages, and will discuss them in more depth below.

**Figure 1:**
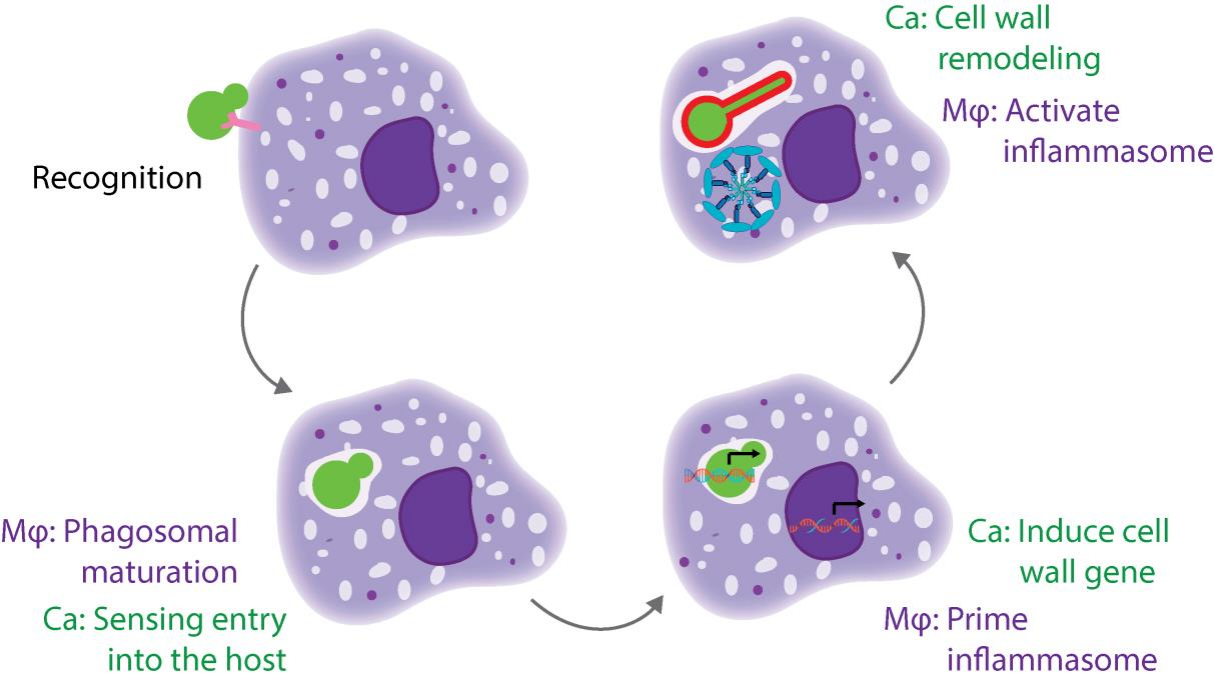
FIGURE 1: A cartoon model of the different stages of interaction between a host macrophage and a *C. albicans* cell. This model highlights the stages that are required for induction of macrophage pyroptosis. Mϕ, macrophage; Ca, *Candida albicans*.

The first step for macrophages is to recognize and phagocytize the fungal cell. A key host receptor that mediates phagocytosis is Dectin-1, a C-type lectin receptor (CLR) that recognizes β-glucan. Mutants with increased β-glucan exposure are more readily phagocytized by macrophages, as in the case of a sextuple mannosyltransferase mutant.

The next stage is that *C. albicans* must sense entry into the host cell, while the host cell phagosome matures. Within the phagosome, the fungus encounters limited nutrients, alterations in osmolarity, and antimicrobial defense mechanisms such as oxidative stress. Our screen identified mutants that are likely to be defective in sensing multiple features of the host environment, including mutants for five genes encoding amino acid permeases and three genes encoding proteins involved in ion transport. These mutants may be unable to recognize entry into the host cell, or they may have defects in growth as they are unable to fully utilize nutrient sources in the phagosome. Altered nutrient availability may explain why we identified *C. albicans* metabolic genes as important for activation of macrophage pyroptosis; depletion of these gene products may impair fungal proliferation within macrophages. However, the robust filamentation of these strains within the macrophage shows that these genes are not simply required for survival. Instead, the defect of these strains may be a reflection of altered pools of metabolic precursors that are necessary for the cell wall remodeling program. We also identified genes that are normally upregulated upon DNA damage; depletion of these gene products may impair cellular responses to the oxidative burst that macrophages deploy as an antimicrobial response.

During the next stage of the interaction, both the fungus and the host need to initiate the transcriptional programs that enable pyroptosis. On the host side, macrophages need to prime the inflammasome by increasing transcription of the inflammasome components, such as NRLP3 and IL-1β. The toll-like receptors (TLRs) are the canonical signal for priming the inflammasome in response to bacterial cues such as lipopolysaccharide (LPS). We used knockdowns of the TLR adaptor MYD88 to show that TLR signaling is also required for pyroptosis in response to fungi. Our analysis also highlighted the importance of C-type lectin receptors (CLRs), as knockdown of the CLR adaptors Bcl10 and Malt1 reduced pyroptosis. We hypothesized that CLR signaling may also be required for inflammasome priming, so we used qRT-PCR experiments in bcl10 knockout macrophages to demonstrate that CLR signaling through Bcl10 is required for inducing *IL1*β and *NLRP3* transcripts in response to fungi. These results implicate both TLR and CLR signaling in inflammasome priming in response to fungi.

For *C. albicans*, the transcriptional response stage needs to integrate multiple signals of host entry to initiate filamentation and cell wall remodeling programs. A major signature from our screen was the Hog1 cascade, where perturbation at any step of this cascade resulted in a decrease in pyroptosis. We used qRT-PCR to show that cells use the Hog1 cascade to transcriptionally regulate cell wall gene expression within macrophages. Many other signaling cascades regulate the transcription of these cell wall genes, such as the Pkc1 MAPK cascade; however, depletion of components of the PKC cascade impairs filamentation and confounds further analysis of pyroptosis.

Finally, the *C. albicans* cell has to expose the trigger for inflammasome activation, and the host cell has to respond and activate the inflammasome. We had previously implicated cell wall remodeling and exposure of glycosylated proteins as a requirement for pyroptosis. In our current study, we also found an enrichment for cell wall genes as important for activation of pyroptosis. One hypothesis is that any alteration in the cell wall would result in a defect in pyroptosis. To test this, we performed a comprehensive analysis of all available *C. albicans* cell wall mutants and their interactions with macrophages. We identified many strains with defects in filamentation, but of the filamentcompetent strains, there was specificity in the gene set required for pyroptosis. This suggested that not all perturbations of the fungal cell result in defects in the induction of pyroptosis. One interesting finding was that the GPI-anchored protein Pga52 was both necessary and sufficient for inducing pyroptosis. This may occur through mannosylation of Pga52, and additional epistasis experiments and substitutions of key Pga52 residues are needed to define the activating epitope.

For the host cell, the mechanisms by which it activates the inflammasome are not fully defined. Different components of the fungal cell wall are recognized by many different CLRs, and CLR signaling through the CARD9 adaptor was previously thought to both prime and activate the inflammasome. As we had demonstrated that the CLRs are required for priming, we initially hypothesized that cell wall remodeling would be required to prime the inflammasome and that the mutant lacking Hog1, with its altered cell wall, would be defective in priming. However, there was no significant difference between the mutant and the wild-type strain in the transcriptional induction of *NLRP3* and *IL1β*. Consistent with this finding, depletion of Pga52 also did not impair priming. To test whether priming induced by the *hog1*Δ*/*Δ mutant was functional, we added a substimulatory dose of nigericin and were able to rescue the pyroptosis defect. This result demonstrated that inflammasome priming and activation are not necessarily coupled in the response to fungi. Moreover, it showed that cell wall remodeling must be required for activating the inflammasome, and not for inflammasome priming.

Our results on the importance of fungal cell wall remodeling also stand in contrast with a previous model that inflammasome activation occurs through rupture of the phagolysosome. To test whether phagolysosomal rupture was indeed required for ASC oligomerization, we used confocal microscopy to visualize the inflammasome and marked the phagolysosome with a LAMP1 antibody or through pulse-chase labeling with sulforhodamine B. In each case, we saw intact phagolysosomal membranes when ASC specks were present, showing that rupture is not a prerequisite for inflammasome activation. The overall activating signal has not been fully defined, and the mechanism by which it moves from the phagosome to the cytosolic NLRP3 inflammasome has yet to be discovered. It will be important to examine additional host pathways and receptors to identify the activating signal.

We can also think beyond the initial host-pathogen interaction and examine what happens at the level of the infected host when pyroptosis occurs. We examined the consequences of altered pyroptosis during systemic infection of mice by quantifying PMN cell recruitment to the kidneys as the cytokines released by pyroptosis are likely to drive inflammation at the site of infection. Wild-type *C. albicans* infections are characterized by a robust neutrophil response. When mice were infected with mutants that fail to activate pyroptosis, we observed primarily mononuclear cell recruitment to the kidneys, which suggests that pyroptosis is a key signal for neutrophil recruitment to the sites of infection.

There are many interesting questions that remain to be answered about fungal activation of host pyroptosis. What is the activating signal in the macrophage that is sensed by the fungal cell that allows for cell wall remodeling? How and why are the filamentation and cell wall remodeling programs coordinated? It is likely that the host requires mechanisms to differentiate between fungi with high pathogenic potential and those that exist in a commensal state. Does this explain why macrophages require priming from both the TLRs and the CLRs? It will also be important to examine the role of fungal activation of pyroptosis during multiple models of disease because depending on the site of infection, the inflammasome can be either essential for clearing infections or a major driver of immunopathology.

